# Neutralizing antibody to VEGF reduces intravitreous neovascularization and may not interfere with ongoing intraretinal vascularization in a rat model of retinopathy of prematurity

**Published:** 2008-02-11

**Authors:** P. Geisen, L. J. Peterson, D. Martiniuk, Ahbineet Uppal, Y. Saito, M. Elizabeth Hartnett

**Affiliations:** 1 Department of Ophthalmology, University of North Carolina at Chapel Hill School of Medicine, Chapel Hill, North Carolina; 2 Carolina Cardiovascular Biology Center, University of North Carolina at Chapel Hill, Chapel Hill, North Carolina

## Abstract

**Purpose:**

To study the effects of a neutralizing antibody to vascular endothelial growth factor (VEGF), given as an intravitreous injection, on intravitreous neovascularization (IVNV) and ongoing vascular development of avascular retina in a rat model relevant to human retinopathy of prematurity.

**Methods:**

Newborn Sprague-Dawley rats were exposed to oxygen fluctuations alternating between 50% O_2_ and 10% O_2_ every 24 h. At postnatal day (p)12, rat pups received intravitreous injections of a neutralizing antibody to VEGF or control nonimmune rat IgG in one eye and were returned to oxygen cycling until p14, at which time they were placed into room air. At p18 (time of maximal IVNV) or p25 (time point in regression), animals were sacrificed. Their retinas were dissected, flat mounted, and stained with Alexa-isolectin for fluorescence microscopy. IVNV was measured as number of clock hours involved in injected VEGF antibody and control eyes. Mean clock hours of IVNV, avascular/total retinal areas and capillary densities within vascularized retinas were determined in injected eyes of control and treatment groups. Mean clock hours of IVNV in fellow noninjected eyes from control and treatment groups were analyzed by Student’s *t*-tests to assess possible crossover effects from systemic absorption of antibody. Eyes from p13 rat pups were sectioned for immunohistochemistry or analyzed for VEGF receptor 2 (VEGFR2) phosphorylation by western blot. Free retinal VEGF at p13, one day following injections, was measured by ELISA.

**Results:**

Neutralizing antibody to VEGF at 25 ng and 50 ng caused a modest but significant inhibition of IVNV compared to IgG injected controls at p18, but only the 50 ng dose decreased IVNV compared to control at p25 (one-way ANOVA p=0.003; posthoc Bonferroni *t*-test p=0.003). Neither dose caused a significant difference in avascular/total retinal area at p18 compared to control. However, at p25, the 50 ng dose caused a significant reduction in avascular/total retinal area compared to the 25 ng dose (ANOVA p=0.038; posthoc Student’s *t*-test p=0.038). There was no difference in avascular/total retinal area between IgG and the 25 ng dose. At p13, qualitative analysis of immunohistochemical sections of retina showed the 50 ng dose of VEGF antibody reduced VEGFR2 phosphorylation within the retina and around blood vessels. Also at p13, there was a significant increase in free intraretinal VEGF protein in eyes that had been treated with 50 ng dose of VEGF antibody compared to IgG injected control (Student’s *t*-test p=0.042). There were no differences in capillary densities in the vascularized retinas between eyes injected with the 50 ng dose of VEGF antibody and IgG control. There was also no difference in weight gain between treated and control groups.

**Conclusions:**

Neutralizing antibody to VEGF at a 50 ng dose caused a significant and sustained reduction in IVNV without interfering with ongoing retinal vascularization in a rat model of ROP, whereas a lower dose of antibody did not. These data also suggest that compensatory regulatory mechanisms may lead to increased VEGF concentration after intravitreous injection of a neutralizing antibody to VEGF. Further study is necessary for safety and for determination of drug dose of VEGF antibody, since dose of treatment appears important and may vary among infants with severe ROP. In this study, survival of already developed retinal capillaries did not appear affected. Neutralizing VEGF by an intravitreous injection of antibody may offer a treatment consideration for severe ROP, which fails current standard of care management.

## Introduction

Retinopathy of prematurity (ROP) is a leading cause of childhood blindness worldwide [1]. An important feature of the pathology in ROP is intravitreous neovascularization (IVNV), which develops at the junction between avascular and vascular retina. The IVNV grows into the vitreous gel rather than into the retina, bleeds and with fibrovascular contraction, leads to retinal detachment and blindness [1,2].

Years ago, a hypothesis was put forth that the hypoxic and avascular retina in diseases like ROP released an angiogenic factor that caused pathologic angiogenesis to develop and appear as IVNV [3-5]. Among several angiogenic factors, vascular endothelial growth factor (VEGF) has emerged as one of the most important in the development of IVNV [6,7]. VEGF is upregulated by hypoxia and ischemia [8,9] and is increased in the serum and vitreous of patients with diseases characterized by IVNV [10]. In addition, IVNV has been reduced in experimental models in which the action of VEGF was inhibited through addition of soluble receptors [11], antibodies to VEGF receptor-2 (VEGF-R2) [12], oligonucleotides [13], or aptamers [14]. In human adults, agents that inhibit the bioactivity of VEGF have dramatically reduced ocular morbidity in several neovascular eye diseases, including diabetic retinopathy and age-related macular degeneration [15-17].

Since the current management for acute severe ROP is ablation of the peripheral avascular retina with laser or cryotherapy [18,19], the question arises whether an agent that inhibits the biologic activity of VEGF would be more effective and less destructive than the current management. A few case series have been reported on short-term effects of anti-VEGF agents in acute ROP [20,21]. However, VEGF is essential for normal retinal vascular development [22-25], and is an endothelial and neuronal survival factor [26,27]. Since retinal vascular development is ongoing in the premature infant, several questions remain before considering treatment of ROP with agents that inhibit the actions of VEGF. First, would inhibition of VEGF reduce IVNV without interfering with ongoing retinal vascularization? A previous study using the mouse model of hyperoxia-induced vasoobliteration and revascularization showed that a neutralizing antibody to VEGF interfered with preretinal endothelial budding but appeared to allow revascularization into the previously hyperoxia-induced obliterated retina [28]. The authors, however, noted that they were unable to measure the area of avascular retina in the mouse model. Also, the mouse model uses high constant oxygen, which is not as relevant to most cases of human ROP in the U.S.today.

Second, would inhibition of VEGF compromise newly developed retinal vasculature or have adverse effects from systemic absorption? To address these questions, we used the Penn “50/10” oxygen-induced retinopathy (OIR) model [29] to test a neutralizing antibody to VEGF (VEGFab), which has a mechanism of action similar to current treatments used in adult eye disease [17].

The oxygen extremes in the Penn 50/10 OIR model [29] were found to be similar to the transcutaneous oxygen levels measured in a premature infant that developed severe ROP [30], as inspired oxygen levels rat pups breathe directly correlate with rat arterial oxygen levels (PaO_2_) [29]. Also, rather than the constant oxygen used in other models [12,31-34], the 50/10 OIR model exposed pups to fluctuations in oxygen, a risk factor for severe ROP [30,35,36]. Finally, the 50/10 OIR model reproducibly and consistently developed IVNV and avascular retina similar in appearance to acute Stage 3 ROP [29,37] and underwent natural regression of IVNV with later vascularization of the previously avascular retina. The outcomes are quantifiable: IVNV at the junction of vascular and avascular retina; the percent peripheral avascular/total retinal area; and the number of capillary junctions within an area of vascularized retina (capillary density). The features of the 50/10 OIR model made it relevant to ROP and useful to evaluate our research hypotheses. We found that of the doses of VEGFab tested, the 50 ng dose sustained inhibition of IVNV and did not interfere with ongoing vascularization of the retina. We also found no adverse effect on the density of newly formed retinal capillaries in vascularized retina or evidence of an adverse effect from systemic absorption. However, one day following intravitreous injection of antibody to VEGF qualitatively reduced intraretinal VEGFR2 phosphorylation and caused an increase in the retinal concentration of free VEGF compared to control.

## Methods

All animal studies complied with the University of North Carolina’s Institute for Laboratory Animal Research (Guide for the Care and Use of Laboratory Animals) and the ARVO Statement for the Use of Animals in Ophthalmic and Visual Research.

### Animal model of retinopathy of prematurity

Litters of 12–14 newborn Sprague-Dawley rat pups, postnatal age 0 (p0), with their mothers (Charles River, Wilmington, MA) were placed into an Oxycycler incubator (Biospherix, New York, NY), which cycled oxygen between 50% O_2_ and 10% O_2_ every 24 h. At p14, the pups were returned to room air for 4 or 11 days [29]. Carbon dioxide in the cage was monitored and flushed from the system by maintaining sufficient gas-flow. The pups developed IVNV at p18 [38] and regression of IVNV with vascularization of the previously avascular retina at p25-p30 [29].

**Figure 1:**
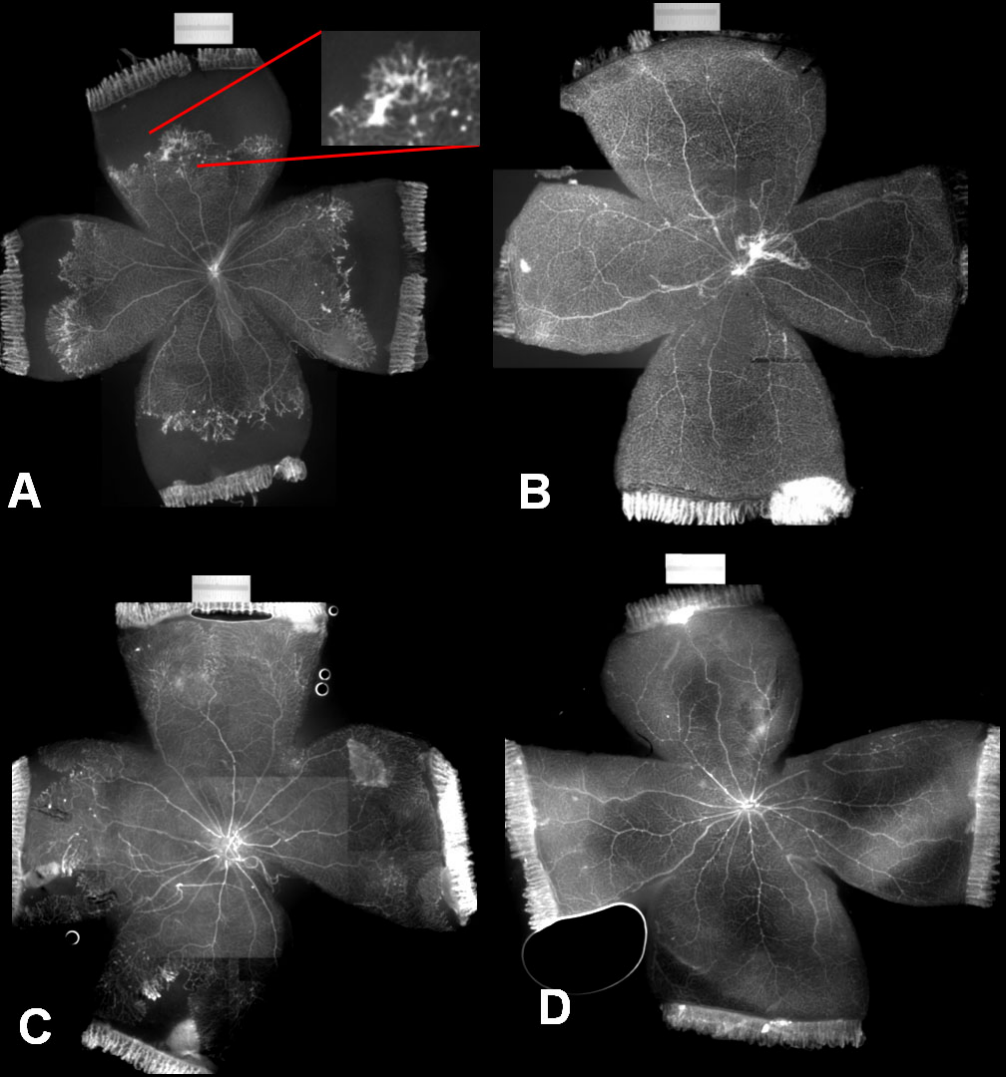
Lectin-stained flat mounts of retinas in pups exposed to oxygen-induced retinopathy or room air at p18 and p25. A: Rat pups exposed to the 50/10 oxygen-induced retinopathy (OIR) model developed IVNV at the junctions of vascular and avascular retina after return to room air (RA). Retinal flat mounts were made and stained with isolectin to reveal the retinal vasculature and intravitreous neovascularization (IVNV) at the junction of vascular and avascular retina at p18 in 50/10 oxygen-induced retinopathy (50/10 OIR)(A) or room air (RA) B: Lectin-stained retinal flat mount from a p18 RA rat pup. C, D: Lectin-stained retinal flat mounts from OIR (C) and RA (D) pups at p25. An example of IVNV has been enlarged for clarity (inset, A).

### Neutralizing VEGF bioactivity

VEGFab, a neutralizing antibody to VEGF_164_ that recognizes rat (R & D Systems, Minneapolis MN) was administered as an intravitreous injection at doses of 25 or 50 ng/μL. Nonimmune rat IgG was used as a control (R & D Systems).

### Intravitreous injections

Rat pups were anesthetized with an intraperitoneal injection of a mixture of 20 mg/kg ketamine and 6 mg/kg xylazine (both from NLS Animal Health, Pittsburgh, PA). A topical anesthetic (0.5% tetracaine hydrochloride) was administered before inserting a 30-gauge needle just posterior to the limbus to avoid lens damage. One µL injections were performed in right eyes using a Hamilton syringe. We then applied 0.5% topical erythromycin ointment (Fougera, Melville NY) to the injected eye. All fellow eyes were not injected. Animals were monitored until recovery (~2 h) and then returned with their mothers to the Oxycycler for two more days. At p14, each litter was removed from the Oxycycler and placed into room air until p18 or p25. All pups were weighed at the times of injection and sacrifice. Mean weights of treated and control pups were determined.

### Dissecting retinal tissue for flat mounting and cryosections

Pups were anesthetized at either p13 for immunohistochemical staining or p18 or p25 for retinal flat mounts by intraperitoneal injection of 60 mg/kg ketamine and 18 mg/kg xylazine. We directly perfused 1.0 mL paraformaldehyde (0.5%) into the left ventricle before euthanasia by intracardiac injection of 50 μl pentobarbital (80 mg/kg). Both eyes were enucleated and fixed in 2% paraformaldehyde for 2 h. Using a modification of the method of Chan-Ling [39], the anterior segments were removed and the retinas with intact ora serratas were dissected and placed into PBS after removal of the hyaloidal vessels and any remaining vitreous. Four incisions were made 90 degrees apart. The retinas were flattened and then placed onto microscope slides. For cryosections, intact fixed eyes with only the cornea, lens, and vitreous removed were put into 30% sucrose/PBS overnight. Each eye was blotted with filter paper to remove excess liquid, soaked in optimal cutting temperature compound (Tissue-Tek, Torrance, CA) and kept at −80 °C for future analysis.

**Figure 2:**
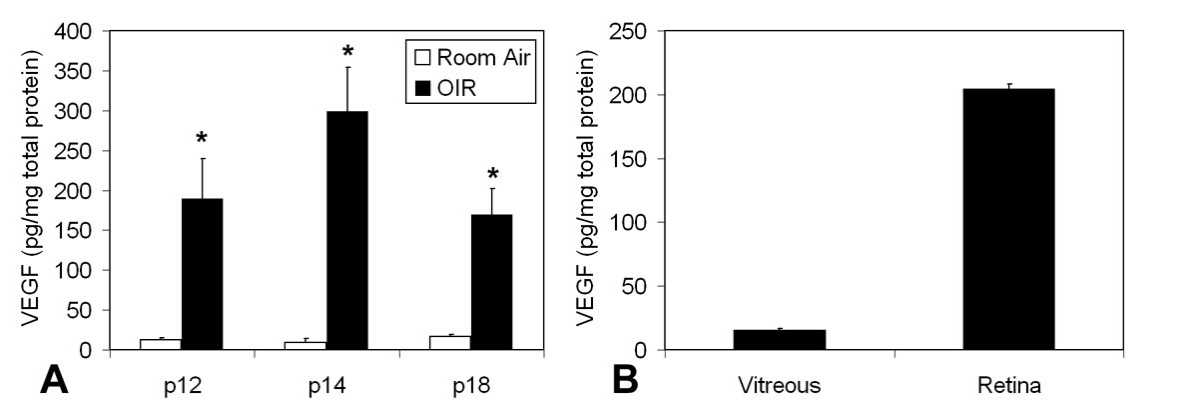
VEGF concentration increased in the 50/10 oxygen-induced retinopathy model compared to room air. A: Vascular endothelial growth factor (VEGF). VEGF concentration was increased in the 50/10 oxygen-induced retinopathy (OIR) model compared to RA at all time points (overall ANOVA p<0.001; post-hoc Student’s *t*-tests * p<0.001). In 50/10 OIR model, VEGF concentration at p14 was greater than at p12 or 18 (ANOVA p<0.001; posthoc Student’s *t*-tests, * p<0.001; n=6 for each time point). B: VEGF concentration was measured by ELISA in the retina (204.5±12.4 pg/mg) and vitreous (15.7±4.3 pg/mg) of p18 OIR-raised rat pups (n=12 for 50/10 OIR and RA).

### Tissue staining

To stain the retinal vasculature, the flattened retinas were permeabilized in ice-cold 70% v/v ethanol for 20 min, then in PBS/1% Triton X−100 for 30 min, and then incubated with 5 μg/ml Alexa Fluor 568 conjugated *G. simplicifolia* (Bandeiraea) isolectin B4 (Molecular Probes, OR) in PBS overnight at 4 °C. Each slide was rinsed three times in PBS, mounted in PBS:glycerol (2:1) with VectaShield (Vector Labs, CA), and protected with a coverslip, which was then sealed with nail varnish. Images of the retinal blood vessels were captured using a Nikon TE2000U inverted microscope (Michael-Hooker Microscopy Facility, University of North Carolina, Chapel Hill) and digitally stored for analysis. Image sections were assembled using methods that maintained the original image dimensions and that did not induce image distortion using Tekmate’s PhotoFit Premium v1.44 (Tekmate, Tokyo, Japan) or with Adobe Photoshop 7.0 (Adobe Systems, San Jose, CA).

### Tissue staining for cryosections

#### Eyes

Eyes frozen in optimal cutting temperature compound were cut into 10-μm sections, adjacent to or within 10 μms of one another. For qualitative comparisons, we placed all labeled serial sections on the same microscope slide to assure equal handling and antibody labeling conditions. Sections were first incubated in PBS/1% Triton X-100 for 30 min. Some retinas were incubated with Alexa Fluor 568-isolectin B4 in PBS for 30 min at room temperature to stain the vasculature. Retinas were washed in PBS three times before they were incubated for 30 min in 3% normal goat serum to block nonspecific binding of the primary antibody. Antiphospho-VEGFR1 (Upstate, Lake Placid, NY) and antiphospho-VEGFR2 (Santa Cruz Biotechnology, Santa Cruz, CA), both polyclonal rabbit antibodies specific for rat protein, were used at a dilution of 1:100. Retinas were incubated for 60 min at room temperature. After three washes in PBS, retinas were incubated for 20 min with a 1:500 dilution of goat antirabbit conjugated with Alexa-488 (Invitrogen, Carlsbad, CA). All retinal cryosections were rinsed three times in PBS, then some were incubated with a 1:5,000 dilution of Hoechst 33342 (Invitrogen) for 15 min, and all were mounted in PBS-glycerol (2:1 with VectaShield; Vector Laboratories). Some sections were stained without the primary antibody as a negative control to observe possible nonspecific binding. On all slides, the coverslips were sealed with nail polish, and images of the sections were captured with a Leica SP2 scanning laser confocal microscope (Leica, Wetzlar, Germany) and digitally stored for analysis. Analysis was qualitative and based on a scale: 0 – none; 1+ weakly present; 2+ strongly present.

### Measurement of intravitreous neovascularization

To determine the extent of IVNV, retinal images from injected and fellow noninjected eyes from experimental and control groups were randomized, labeled, and analyzed for IVNV clock hours. Two masked reviewers performed all analyses. The presence of IVNV was determined with a technique adapted from those used in clinical trials [18] and animal model determination [40]. Flat mounts were divided into 12 clock hours of approximately equal area using Adobe Photoshop, assessed for the presence of IVNV [18,40], and assigned a number (0 to 12) based on the number of clock hours exhibiting IVNV.

**Figure 3:**
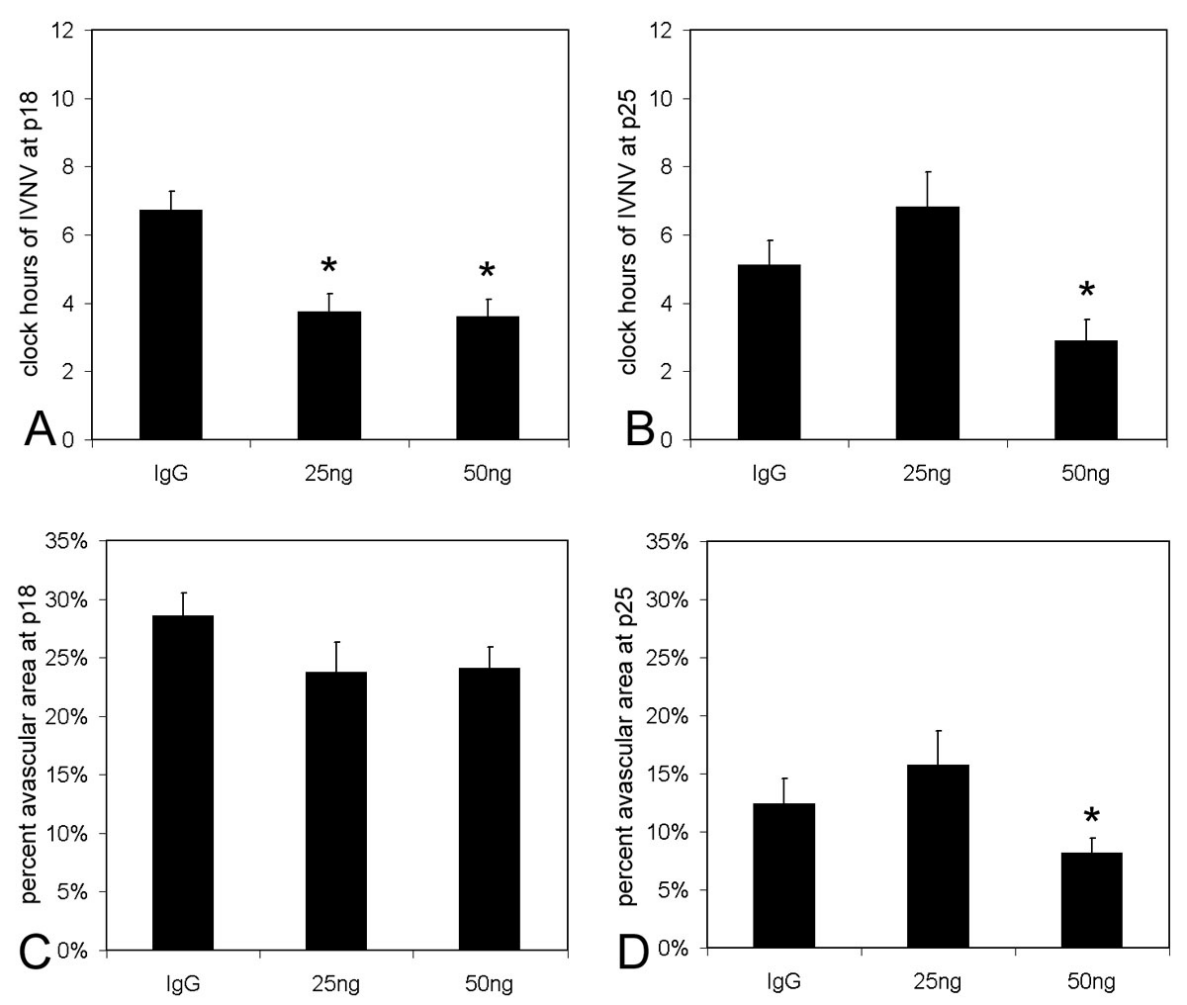
Clock hours of intravitreous neovascularization and avascular/total retinal areas of lectin-stained retinal flat-mounts from rat pups in 50/10 oxygen-induced retinopathy after intravitreous injection of neutralizing antibody to vascular endothelial growth factor (VEGFab) or control nonimmune rat IgG. A: Mean clock hours of intravitreous neovascularization (IVNV)at p18 were significantly decreased by injection of either 25 ng or 50 ng VEGFab compared to IgG control (ANOVA p<0.001; * posthoc Student’s *t*-tests p<0.001 compared to IgG). B: Mean clock hours of IVNV at p25 were significantly decreased by injection of 50 ng VEGFab compared to IgG control (ANOVA p=0.003; * posthoc Student’s *t*-test p=0.003). C: Peripheral avascular/total retinal area was no different in retinas treated with either VEGFab dose compared to IgG control at p18 (ANOVA p=0.238). D: At p25, the overall ANOVA for peripheral avascular/total retinal area was significant (p=0.038). Posthoc testing showed the 50 ng dose of VEGFab was significantly decreased compared to the 25 ng dose (* posthoc Student’s *t*-test, 25 ng versus 50 ng, p=0.038). However, neither dose of VEGFab was significantly different to control IgG.

### Analysis of peripheral avascular areas and quantification of capillary density

Digitized images of the total retinal area and peripheral avascular areas were measured (ImageTool v.3, University of Texas, San Antonio, TX). The peripheral avascular area was expressed as a percentage of the total retinal area for experimental and control eyes. Central capillary density was quantified as the summation of capillary junctions (crossings) within four equal square areas, each 0.16 mm^2^, in each of the four quadrants of the vascularized retina and expressed as number of junctions per 0.64 mm^2^ using ImageTool [37].

### Fresh tissue preparation

Animals were euthanized with an 80 mg/kg intraperitoneal injection of pentobarbital. Both eyes were enucleated, and the retinas were isolated under a dissecting microscope in similar fashion as for flat mounting, except that the ora serratas were removed. The tissue was placed into modified radioimmuno precipitation assay (RIPA) buffer (20 mM Tris base, 120 mM NaCl, 1% Triton X-100, 0.5% sodium deoxycholate, 0.1% SDS, 10% glycerol) for ELISA and frozen at −20 °C until analysis. For one litter (12 rat pups), the cornea and lens were removed bilaterally, and the vitreous then was collected from the eye-cup to avoid contamination from blood before removal of retinas for analyses. Vitreous samples from each animal were pooled in pairs, put into RIPA and analyzed for VEGF by ELISA.

**Figure 4:**
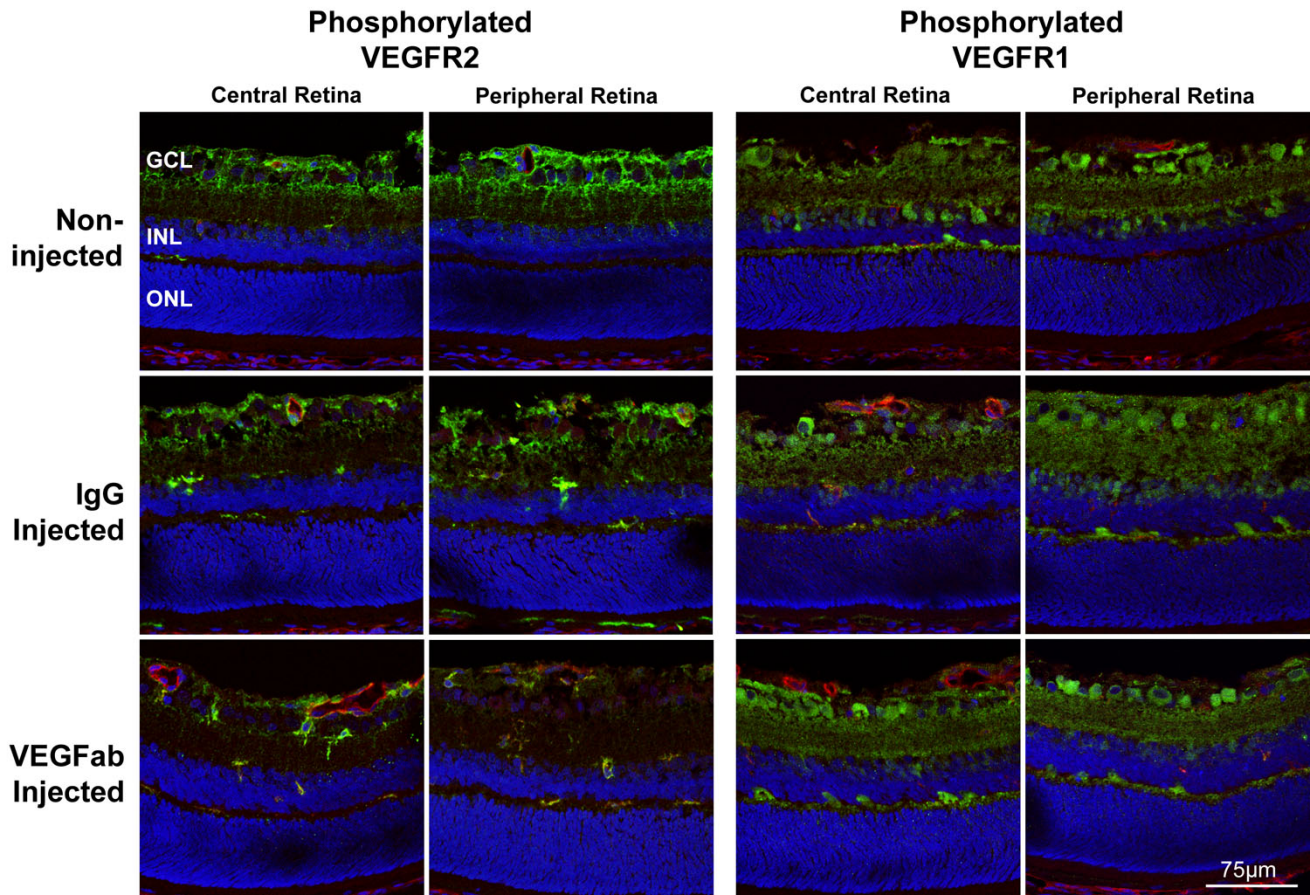
VEGFR2 phosphorylation was decreased by injection of 50 ng VEGFab. Vascular endothelial growth factor receptor 2 (VEGFR2) phosphorylation was noted mainly at the ganglion cell/nerve fiber layer and superficial and deep retinal vessels in noninjected and IgG injected controls (2+, upper left). Phosphorylation of VEGFR2 was reduced after injection with 50 ng VEGFab (1+, lower left). VEGFR1 phosphorylation was noted at the ganglion cell/nerve fiber layer and outer plexiform layer in noninjected and IgG injected controls (2+, upper right). No change noted in 50 ng VEGFab-injected eyes (2+, lower right). All injections were performed at p12 and eyes removed at p13. Green indicates phosphorylated VEGFR1 or phosphorylated VEGFR2; red indicates lectin-stained vasculature. Central retina is toward the optic nerve, and peripheral retina is within vascularized retina near the junction with avascular area.

### Western blot for phosphorylation of VEGF receptors 1 and 2

Freshly dissected unfixed retinal tissue immersed in modified RIPA buffer with a 1:100 protease inhibitor cocktail (Sigma, St. Louis, MO) were homogenized, and lysates centrifuged at 13,000xg for 15 min at 4 °C. The supernatants were collected, and total protein was quantified by bicinchoninic acid (BCA) assay according to the manufacturer’s protocol (Pierce, Rockford, IL). Next 75 μg protein samples were immunoprecipitated overnight with 2 μg of a polyclonal antibody to VEGFR-2 (Santa Cruz Biotechnology). The immune complexes were then bound to Sepharose G Protein beads for 1 h before they were washed three times with RIPA buffer. The protein-sepharose complex was eluted in 2x sample buffer (Laemmli sample buffer with 5% β-mercaptoethanol), boiled and run by SDS–PAGE. After transfer to polyvinylidene fluoride (PVFD) (Millipore, Billerica, MA) using standard protocols, the blots were blocked in 5% bovine serum albumin/Tris-buffered saline with Triton X-100 (BSA/TBST) for 1.5 h at room temperature, then incubated in a 1:1000 dilution of antiphospho-VEGFR-2 (Santa Cruz Biotechnology) antibody overnight with gentle agitation at 4 °C. Blots were washed four times in TBST buffer then incubated 1 h with horseradish peroxidase (HRP)-conjugated secondary antibody and washed with TBST. Immunoreactive proteins were detected with Immobilon Western chemiluminescence (Millipore) and analyzed with Un-scan-it v6 (Silk Scientific, Orem, UT).

### ELISA of VEGF

Frozen vitreous and retinal tissue stored in modified RIPA buffer with protease inhibitor cocktail were homogenized, thawed, and subjected to centrifugation at 13,000x*g* for 10 min at 4 °C. Total protein was quantified using a BCA assay according to the manufacturer’s protocol (Pierce). Supernatants were then assayed without dilution in duplicate using a commercially available ELISA kit, raised against rat VEGF_164_ isoform (R& D Systems).

### Statistical analysis

For individual experiments and as groups based on condition and time point of analysis, the magnitude of within subject effects was assessed by Student’s *t*-tests that compared clock hours of IVNV from noninjected eyes of treatment and control groups. In cases with more than two treatment groups analyzed, one-way ANOVA was performed with posthoc Bonferroni correction. Each group compared in flat mount analysis had at least 12 or more pups from at least two litters. The magnitude of treatment effect on bodyweight gain was assessed by comparison of the mean bodyweights between treated and control groups assayed at the same time point by Student’s *t*-tests. For all comparisons, an alpha level of <0.05 was used as the criterion of significance.

**Figure 5:**
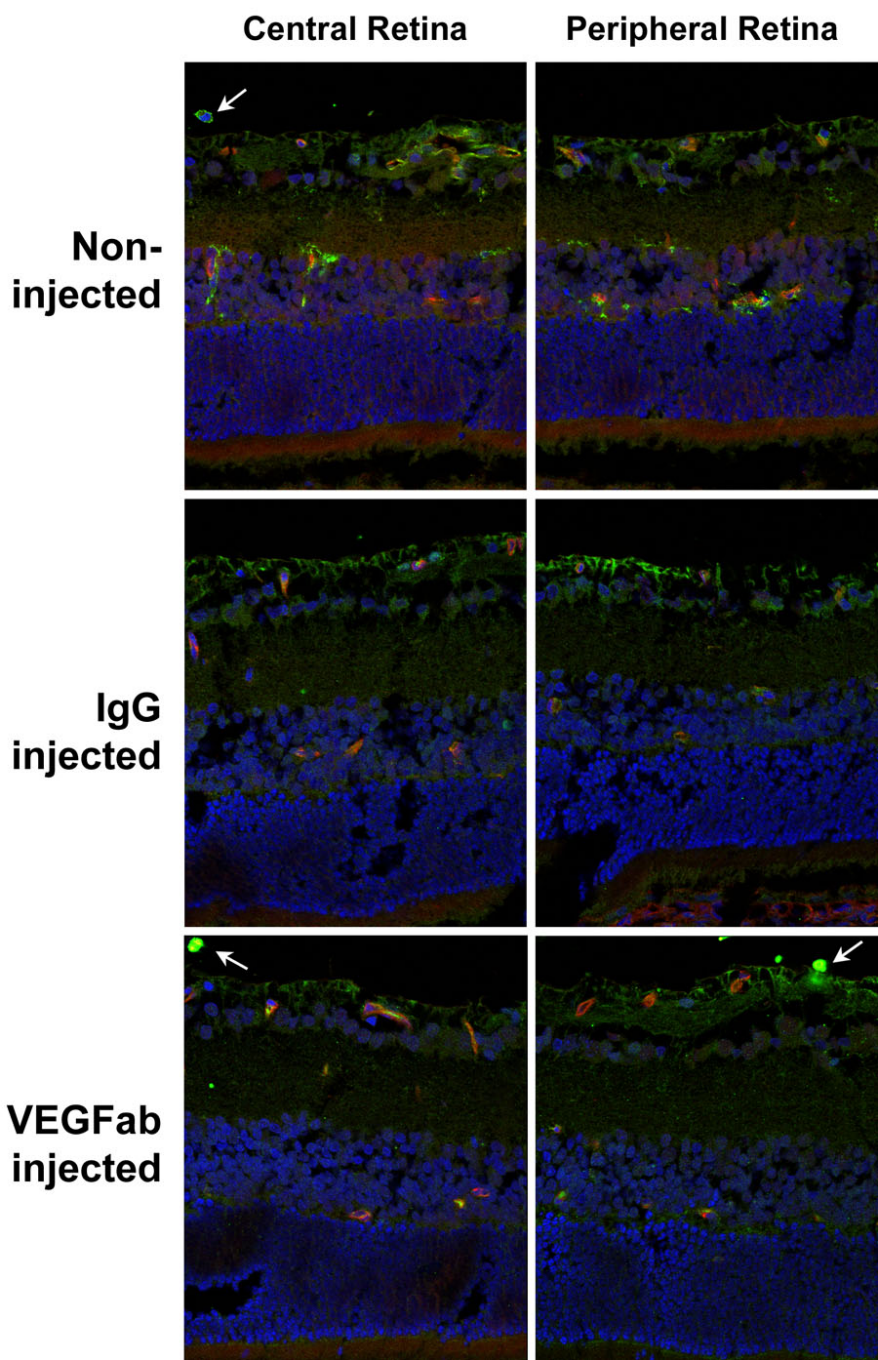
Phosphorylation of VEGFR2 at p18. Phosphorylation of vascular endothelial growth factor receptor 2 (VEGFR2) in lectin-stained IVNV (arrow) in p18 in noninjected, IgG injected and 50 ng VEGFab injected eyes. There was no qualitative difference in phosphorylation between groups.

## Results

### Model of retinopathy of prematurity (50/10 oxygen-induced retinopathy model)

From all experiments, mean clock hours of IVNV and percent avascular retina were determined for the fellow noninjected eyes of pups exposed to the 50/10 OIR model. At p18, 8 ±0.9 (mean±S.E.) clock hours of IVNV and 19.3% ±4.0% (mean±S.E.) avascular/total retinal area were present. At p25, 4 ±1.0 (mean±S.E.) clock hours of IVNV and 7.8% ±1.9% (mean±S.E.) avascular/total retinal area were present. In comparison, flat mounts for room-air-raised pups at p18 and p25 had no IVNV and had fully vascularized retinas (Figure 1A-D). Others have reported the time point for maximum IVNV in the 50/10 OIR model to be p20 [38]. However, we measured IVNV clock hours from 2 noninjected litters of pups exposed to the 50/10 OIR model and assayed at either p20 or p18 and found no difference in mean values [t test, p=0.2059; mean clock hours 5.1 +3.7 (p20, n=12) and 7.07+2.8 (p18, n=12)]. For all subsequent experiments, we used p18 as the time point to analyze IVNV and p25 as a time point during regression of IVNV and ongoing vascularization of previously avascular retina.

We previously found that VEGF_164_ mRNA was the only isoform that was upregulated by repeated oxygen fluctuations, a risk factor for severe ROP [41]. We therefore determined the effect of fluctuations in inspired oxygen on VEGF protein measured by ELISA in whole retinas at different time points in the 50/10 OIR model. The ELISA kit uses an antibody raised against full length VEGF_164_. Although this assay does not discriminate among the VEGF isoforms, the most likely isoform is VEGF_164_, because we previously found it to be the predominant isoform expressed at the time of maximum IVNV in the 50/10 OIR model [41]. VEGF protein was significantly increased in the 50/10 OIR model compared to room air at p12, p14, and p18 (ANOVA test p<0.001; Figure 2A). In addition, posthoc Bonferroni analysis revealed a significant increase in VEGF at p14 compared to either p12 or p18 (p<0.001; Figure 2A), similar to that reported by others using this model [38]. We also measured vitreous VEGF at p18 as 15.7 ± 1.25 pg/mg total protein (mean±S.E.) (Figure 2B), which was at the lower limits of detection for the ELISA assay.

### Neutralizing VEGF reduces intravitreous neovascularization while permitting vascularization of the previously avascular retina

Because VEGF protein peaked at p14 in the 50/10 OIR model, we chose p12 (following a 10% O_2_ cycle) as the time point to inject VEGFab or control nonimmune rat IgG. Pups were then returned to cycling until p14, at which time they were placed into room air until analysis. At p18, the time point of maximal IVNV, both the 25 ng and 50 ng doses of VEGFab caused a significant reduction in clock hours of IVNV compared to respective concentrations of IgG (ANOVA p<0.001, posthoc Bonferroni *t*-tests p<0.001 compared to IgG, Figure 3A). Since there was no difference in avascular/total retinal area in clock hours of 25 ng and 50 ng IgG injected groups, data was collapsed into one IgG control group. There was no significant difference in avascular/total retinal area between VEGFab-injected and control groups at p18 (ANOVA, p=0.238, Figure 3C).

At p25, however, only the 50 ng VEGFab dose sustained the inhibitory effect on IVNV (ANOVA p=0.003; posthoc Student’s *t*-test p=0.003, Figure 3B). Furthermore, eyes injected with 25 ng VEGFab had a slight but insignificant increase in the number of clock hours of IVNV compared to control, suggesting that the 25 ng dose may have been inadequate to sustain an inhibitory effect (Figure 3B). The overall ANOVA for mean avascular/total retinal area of eyes injected with any VEGFab dose was significant, and there was a significant decrease in avascular/total retinal area in the 50 ng VEGFab group compared to 25 ng VEGFab group. However, there was no difference between either dose of VEGFab and control IgG at p18 (Student’s *t*-test, p =0.133) or p25 (Student’s *t*-test, p =0.934).

Mean capillary densities measured from vascularized retina of pups whose eyes had been injected with 50 ng VEGFab were not different from those injected with control IgG, (p18, Student’s *t*-test p=0.133; p25, Student’s *t*-test p=0.934). Weight gain of pups from p12 (following a 10% O_2_ cycle) to p18 or from p12 to p25 whose eyes were injected with 50 ng VEGFab were also no different compared to respective measurements from control injected pups (Student’s *t*-tests p>0.05). There was also no apparent cross over effect from systemic absorption of either dose of VEGFab when mean clock hours of fellow noninjected eyes of experimental groups from p18 and p25 were compared to those of respective control groups (Student’s *t*-tests all p>0.05).

### Effects of neutralizing vitreous VEGF on VEGF and VEGF receptor phosphorylation in the retina

**Figure 6:**
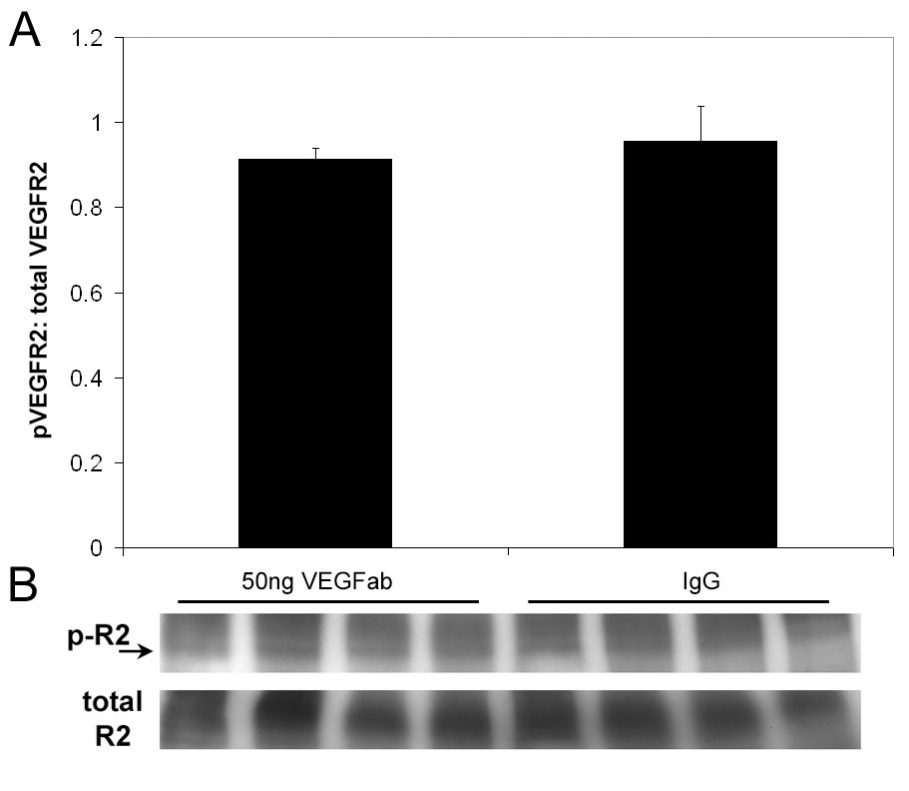
Phosphorylated VEGFR2 by Western blot in 50 ng neutralizing antibody to vascular endothelial growth factor and IgG injected eyes. No difference in retinal vascular endothelial growth factor receptor 2 (VEGFR2) phosphorylation to total VEGFR2 from eyes injected with either 50 ng neutralizing antibody to VEGF (VEGFab) or control IgG at 12 and assayed at 13 (p=0.648, Student’s *t*-test).

To determine the effects of an intravitreous injection of neutralizing antibody to VEGF on retinal VEGF and VEGF signaling through its receptors, VEGFR1 and VEGFR2, we performed immunohistochemical staining and western blot analysis on eyes that had been injected with 50 ng VEGFab or control nonimmune IgG. Cryosections were taken at p13 and were immunolabeled with antibodies to phosphorylated VEGFR-1 or phosphorylated VEGFR-2. Sections were taken centrally (toward the optic nerve) and peripherally within vascularized retina near the junction with avascular retina. In general, in both noninjected and IgG-injected eyes, VEGFR-2 phosphorylation was present mainly in the ganglion cell and nerve fiber layers (2+) and around superficial and deep retinal vessels (2+), whereas VEGFR-1 phosphorylation was present in the ganglion cell (but not nerve fiber layer) and in the outer plexiform layer (2+) (Figure 4). VEGFR-2 phosphorylation was reduced in eyes injected with VEGFab compared to control (1+) at p13 (Figure 4). There was no change observed in VEGFR1 phoshorylation from cryosections among treated control and non-injected eyes (Figure 4). At p18, there was reduced VEGFR2 phosphorylation in the ganglion cell/nerve fiber layer regions in all groups (1+) compared to p13 (Figure 5). Phosphorylation (2+) remained strong in blood vessels and in IVNV at p18 (arrow, Figure 5). There was no difference in VEGFR2 phosphorylation between noninjected, IgG injected, and 50 ng VEGFab injected eyes at p18. Despite the findings on immunohistochemistry, we were unable to detect a significant difference in phosphorylated to total VEGFR2 in whole retinas analyzed by western blot from VEGFab injected (50 ng) and control IgG injected eyes at p13 (Figure 6).

**Figure 7:**
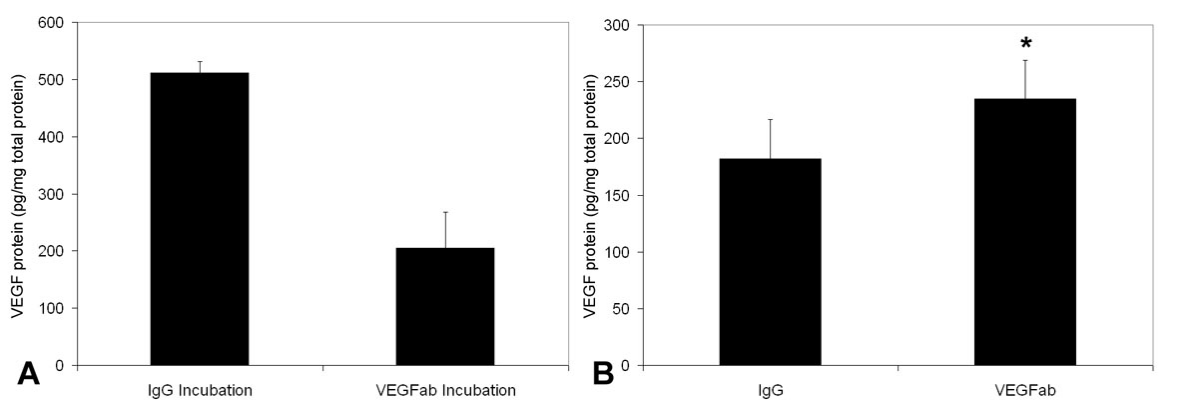
Unbound VEGF. A: Retinas from 50/10 oxygen-induced retinopathy (OIR) noninjected pups at p14 were incubated overnight in either 1 µg neutralizing antibody vascular endothelial growth factor (VEGFab) or IgG. Incubation with VEGFab reduced detectable free vascular endothelial growth factor (VEGF) compared to control IgG. B: Eyes in 50/10 OIR injected with 50 ng VEGFab at p12 showed significantly increased free VEGF in the retina at p13 compared to those injected with IgG (n=5). Asterisk (*) indicates p=0.042 based on Student’s *t*-test.

To determine the effect of an intravitreous injection of VEGFab on free VEGF concentration within the retina, we performed ELISA assays in retinas that had received 50 ng VEGFab or nonimmune IgG. We first determined if antibody-bound VEGF was detectible by ELISA testing. We incubated the same amount of protein from p14 50/10 OIR noninjected retinas with either 1 µg VEGFab or IgG overnight and measured the free VEGF within the samples using an ELISA. We found a decrease in free VEGF measured by ELISA in VEGFab-treated retina compared to control treated retina, providing support that ELISA does not detect VEGFab-bound VEGF (Figure 7A). We then measured free retinal VEGF at p13 in eyes from pups exposed to the 50/10 OIR model and that had received intravitreous injections of 50 ng VEGFab or IgG control injections at p12. VEGF was increased in the eyes that had been injected with 50 ng VEGFab (mean±S.D.: 234.7±34.2) compared to those injected with IgG (mean±S.D.: 182.2±34.4) (Figure 7B) (p=0.042, Student’s t-test). Free vitreous VEGF was below the detectible limits of the ELISA in both groups.

## Discussion

Since the 50/10 OIR model consistently develops IVNV and naturally undergoes regression of IVNV followed by vascularization of previously avascular retina, we were able to test the effects of neutralizing VEGF with antibody on these quantifiable outcomes by analyzing two different time points. We found that neutralizing VEGF with an intravitreous injection of 50 ng of VEGFab administered at p12 caused a significant reduction in IVNV at p18. Furthermore, this dose sustained the effect at p25 and did not interfere with ongoing retinal vascularization. However, the lower dose of antibody did not sustain the inhibitory effect on IVNV.

Antibodies generally are cleared from that eye over several days–on average 5.6 days in monkey vitreous [42,43]. Since vitreous VEGF concentration at p18 was approximately 1/10 of the retinal VEGF (Figure 2B), we propose that an adequate dose of VEGFab may neutralize sufficient vitreous VEGF without severely interfering with intraretinal signaling necessary for ongoing retinal vascular development [23,24,44]. If the concentration of antibody binds both retinal and vitreous VEGF sufficiently to cause vitreous VEGF bioactivity to be effectively zero, then it is possible a compensatory increase in intraretinal VEGF protein, as we found, may promote intraretinal vascularization. If the level of antibody is too low, then both IVNV and intraretinal vascularization would be reduced, but chemoattractive forces in both vitreous and retina may be insufficient to inhibit, but only slow retinal vascularization. Indeed, we found a slight, although insignificant, increase in avascular retina compared to IgG control at p25. Still the avascular retinal area at p25 was less than that at p18.

There is increasing evidence that the role of VEGF in retinal vascular development and oxygen stresses is complex. It has been reported that, a front of migrating cells, e.g., astrocytes in cat [24] or angioblasts in dog [45], sense physiologic hypoxia and express VEGF. The ensuing endothelial cells are attracted to VEGF and migrate to create blood vessels [24]. More recently, it has been found that VEGF concentration may regulate endothelial cell division rate [46,47], but the presentation of VEGF, as in a gradient, regulates endothelial tip cells at the migrating front and direct the growth of endothelial cells [48]. Furthermore, there is evidence that a gain in VEGF signaling through VEGFR2 can cause disoriented endothelial daughter cell divisions rather than orderly angiogenesis [49]. In immunohistochemical qualitative assessment of immunohistochemical sections taken from eyes one day after injections, we found that, compared to control or noninjected eyes, 50 ng of VEGFab appeared to reduce but not entirely inhibit signaling of VEGFR2, the receptor believed most associated with angiogenic processes [50]. Although we were unable to confirm the reduction in VEGFR2 phosphorylation by western blot analysis, we suspect that analyzing whole retinas may dilute effects seen in a small percentage of cells. VEGFR1 phosphorylation did not appear to be affected. This may have been because VEGFR1 has greater affinity for VEGF [50] and would have bound free VEGF not associated with VEGFab. Effects from VEGFab on intraretinal VEGFR2 signaling appeared to have resolved by p18. We also found that free VEGF in the retina measured one day after an injection of 50 ng VEGFab was increased compared to control. Possibly, this represents a compensatory effect and may partly explain the insignificant but increased number of clock hours of IVNV at p25 in eyes injected with 25 ng of VEGFab when compared to Ig G control.

The VEGFab used in this study is greater than 78kD molecular weight, which is the limit above which diffusion beyond the inner plexiform layer becomes extremely slow [43,51]. From our immunohistochemical sections, much of the reduction in VEGFR2 phosphorylation appeared to be in the region of the ganglion cell/nerve fiber layers. However, there is experimental and clinical evidence that antibodies of higher molecular weight, including the humanized mouse monoclonal antibody to VEGF (bevacizumab), penetrate into the retina and can affect signaling in deeper retinal layers [52].

Several papers have shown immunolabeling of VEGF receptors but not phosphorylation, in developing retinal vessels and in IVNV-like vessels or endothelial buds after oxygen stress [12,53-55]. VEGFR2 immunoreactivity was also reported to be strong within IVNV in a beagle model of OIR and weak within newly forming intraretinal vessels during normal development [12]. In addition, VEGF and its receptors were found within neural retina [56-58], mainly the ganglion cells, astrocytes, and Mueller cells. In development, VEGF receptor inhibitors led to a reduction in the thickness of the retina and in the ganglion cell layer [54]. We report that activation of VEGFR2 signaling was associated with intraretinal and intravitreous blood vessels and within the ganglion cell and nerve fiber layers of retinas from pups exposed to the 50/10 OIR model. VEGFR1 phosphorylation was associated mainly with the ganglion cell and outer plexiform layers. We propose that inhibition of VEGFR2 signaling by VEGFab occurred in developing vessels, and within Mueller cell processes, astrocytes, and ganglion cells at p13, and was resolved by p18. Definitive confirmation of what cells are affected will require colabeling of retinal sections in future studies. Although reduced VEGFR2 phosphorylation was associated with decreased IVNV in our experiments, further study is needed to determine if there are significant effects on neuronal and glial cells, particularly if higher doses of VEGFab or prolonged and repeated doses are considered. However, ganglion cells treated with the VEGF antibody, bevacizumab, were not reported to have reduced viability in vitro [59].

Although we found a modest decrease in IVNV compared to control, it would represent a clinically significant outcome in reducing the risk of poor vision in preterm infants with ROP [19]. Many studies have shown that blocking other signaling pathways can inhibit IVNV more completely [60-62]. However, in ROP, aggressive angiogenic inhibition is not desired, because ongoing retinal vascular development may both lead to improved visual function and reduce the hypoxic stimulus for pathologic IVNV.

The effects of inhibiting VEGF on pathologic IVNV and its presumed stimulus, the avascular retina, are relevant questions when considering anti-VEGF strategies for ROP in which it is undesired to inhibit retinal vascular development but necessary to prevent or treat IVNV. Our data suggest that an intravitreous antibody to neutralize VEGF may be effective and safe, but dose appears important. Determining an effective dose in individual infant eyes is difficult because vitreous VEGF protein produced by the hypoxic retina may vary in separate eyes depending on the zone of ROP--i.e., the extent of avascular retina. Currently, vitreous VEGF measurements cannot be obtained safely in human infants with stage 3 ROP. In our study, measuring vitreous VEGF was not always possible in the animal model, because the concentration of VEGF in the vitreous was at the lower limits of detection by ELISA. Very high concentration or slow release formulations of an anti-VEGF antibody may carry a risk to retinal neurons and also theoretically inhibit ongoing retinal vascular development [22-24,26,27]. In addition, too low a dose of anti-VEGF antibody may not be effective or require repeated injections, which increase risk. Furthermore, unlike in the 50/10 OIR model, human infants with ROP do not always undergo disease regression, in part, because of the effect of other factors [1].

When administering an intravitreous injection of any drug into an infant eye, the high vitreous/blood volume of the premature infant to the adult must be considered. The resultant drug concentration from absorption into the systemic circulation is greater in the infant than that in adult and may cause systemic effects [37]. Systemic anti-VEGF agents used in metastatic colon or renal cell carcinoma have been reported associated with several serious effects, including hypertension and vascular events [63-65]. In this study, compared to control injected eyes, we found no adverse effect on weight gain or on outcomes in the fellow eyes, suggesting little effect from systemic absorption at the time points we analyzed. Nor did we find an adverse effect on capillary densities of newly vascularized retina within the treated eyes. Although questions remain, in certain severe forms of ROP with poor outcomes [66,67], use of anti-VEGF agents should be studied and considered.
